# Isolation and Molecular Characterization of Amniotic Fluid-Derived Mesenchymal Stem Cells Obtained from Caesarean Sections

**DOI:** 10.1155/2017/5932706

**Published:** 2017-10-31

**Authors:** Lucas-Sebastian Spitzhorn, Md Shaifur Rahman, Laura Schwindt, Huyen-Tran Ho, Wasco Wruck, Martina Bohndorf, Silke Wehrmeyer, Audrey Ncube, Ines Beyer, Carsten Hagenbeck, Percy Balan, Tanja Fehm, James Adjaye

**Affiliations:** ^1^Institute for Stem Cell Research and Regenerative Medicine, Heinrich Heine University Düsseldorf, Moorenstr. 5, 40225 Düsseldorf, Germany; ^2^Department of Obstetrics and Gynaecology, Heinrich Heine University Düsseldorf, Moorenstr. 5, 40225 Düsseldorf, Germany

## Abstract

Human amniotic fluid cells are immune-privileged with low immunogenicity and anti-inflammatory properties. They are able to self-renew, are highly proliferative, and have a broad differentiation potential, making them amenable for cell-based therapies. Amniotic fluid (AF) is routinely obtained via amniocentesis and contains heterogeneous populations of foetal-derived progenitor cells including mesenchymal stem cells (MSCs). In this study, we isolated human MSCs from AF (AF-MSCs) obtained during Caesarean sections (C-sections) and characterized them. These AF-MSCs showed typical MSC characteristics such as morphology, *in vitro* differentiation potential, surface marker expression, and secreted factors. Besides vimentin and the stem cell marker CD133, subpopulations of AF-MSCs expressed pluripotency-associated markers such as SSEA4, c-Kit, TRA-1-60, and TRA-1-81. The secretome and related gene ontology (GO) terms underline their immune modulatory properties. Furthermore, transcriptome analyses revealed similarities with native foetal bone marrow-derived MSCs. Significant KEGG pathways as well as GO terms are mostly related to immune function, embryonic skeletal system, and TGF*β*-signalling. An AF-MSC-enriched gene set included putative AF-MSC markers *PSG5*, *EMX-2*, and *EVR-3*. In essence, C-section-derived AF-MSCs can be routinely obtained and are amenable for personalized cell therapies and disease modelling.

## 1. Introduction

Recent human AF research has shown that stem cells from the first and second trimester can be collected during amniocentesis (an invasive method of prenatal diagnosis of chromosomal abnormalities and foetal infections) [1]. The therapeutic potential including *in vitro* characterization of human amniotic fluid-derived stem cells (AFSCs) was first reported by the Atala group [2]. Because of their low immunogenicity, anti-inflammatory properties, and high proliferative and differentiation capacity *in vitro*, AFSCs are amenable for clinical application and tissue engineering. Furthermore, they lack carcinogenesis after transplantation in nude mice and have the ability to create embryoid body-like structures after specific treatments. Their possible origin from epiblast, demonstrated by the presence of common features with primordial germ cells, is also under discussion [2, 3]. The AFSC populations are heterogeneous in nature, of foetal-derived-differentiated and undifferentiated progenitor cells [4]. In 1993, Torricelli and coworkers first reported a subpopulation of hematopoietic progenitor cells in AF [5]. Interestingly, in 2003, it was reported that a small subpopulation of AFSCs expresses the pluripotency-regulating marker, octamer-binding transcription factor 4 (OCT4) [6]. Later, Moschidou coworkers reported that AFSCs isolated from the first trimester express other pluripotent stem cell-associated markers such as NANOG, sex-determining region Y-box 2 (SOX2), Krüppel-like factor 4 (KLF4), stage-specific embryonic antigen-4 (SSEA4), CD133, and c-Kit [7, 8]. Their self-renewal capabilities were also confirmed, thus indicating that AFSCs are of high plasticity and easily reprogrammable as our previous studies demonstrated [9, 10]. At the transcription level, it has also been shown that a subpopulation of AFSCs has high overlap with human ESCs as they share about 82% of transcriptome identity [11]. Additionally, AFSCs were found to be paracrine active as their conditioned media contain cytokines which have a profound effect on vasculogenesis, angiogenesis, and osteogenesis [12–14]. AFSCs have the potential for use in clinical applications as shown for example by keratinocyte differentiation and subsequent improvement of wound healing [15].

Mesenchymal stem cells (MSCs) are multipotent stromal stem cells [16, 17]. Morphologically, they are fibroblast-like and spindle-shaped cells. *In vitro*, these clonogenic cells easily adhere to plastic surfaces and have high-replicative capacity [17, 18]. Several sources are reported from adult and foetal tissues from which these types of MSCs can be obtained, for example, bone marrow (BM) and adipose tissue [19] and extraembryonic tissues such as umbilical cord blood [20, 21] and placental tissues such as amnion and decidua and furthermore from second and term amniotic fluid [22]. *In vitro* and *in vivo* MSCs differentiate into mesodermal cell types such as fibroblasts, osteoblasts, chondrocytes, and adipocytes [16, 23]. The International Society for Cellular Therapy (ISCT) postulated that for transplantation and cellular therapy, MSCs should not differentiate into blood cells and therefore not express any markers of hematopoietic lineage such as the surface markers CD14, CD34, and CD45. In contrast to this, bone marrow MSCs should express CD73, CD90, and CD105 referring to their minimal characterization criteria [24]. MSCs have been widely used for therapies such as graft versus host disease, precisely in over 700 clinical trials till date (https://clinicaltrials.gov). The frequency and differentiation capacity as well as proliferation potential from BM-MSCs has been shown to decrease with age [25].

A subpopulation of AFSCs with mesenchymal characteristics has been isolated from second and third-trimester AF and thus referred to as amniotic fluid mesenchymal stem cells (AF-MSCs). They were isolated based upon their plastic adherence and similar cell surface marker composition as MSCs from other sources. Furthermore, they were also able to differentiate into bone, cartilage, and fat cells *in vitro* [23, 26–28]. Various studies have shown that these AF-MSCs also express OCT4 [27, 28]; however, this is still controversial since no one has yet defined the self-renewal function of OCT4 in AF-MSCs as has been shown in human embryonic stem cells [29].

AF-MSCs are advantageous in terms of developmental stages but problematic with respect to the invasiveness of the collection procedures—amniocentesis and foetal infections. Therefore, C-section-derived AF could be an alternative source for these cells. However, the amniotic fluid is merely discarded during this procedure that is why few studies have isolated AFSCs at this stage of gestation. The question remains as to whether full-term AF harbours AF-MSCs of similar potency as cells obtained in the first and second trimesters of pregnancy.

In this study, we characterized human AF-MSCs obtained from C-sections (third trimester) and tested their multilineage differentiation capacity *in vitro*, immunophenotype, expression of mesenchymal markers, multipotency markers, transcriptome, and their secretome. Our data suggests that AF obtained from C-sections may represent a promising source for stem cells of mesenchymal origin. Presently, the most common source of MSCs is the bone marrow (BM). However, harvesting and processing of BM-MSCs have major drawbacks and limitations. Thus, it is significant that AF collected during C-sections is an alternative source of AF-MSCs that are immature and possess high plasticity making them useful for clinical applications.

## 2. Materials and Methods

### 2.1. Preparation of Amniotic Fluid

Three amniotic fluid samples from healthy human donors were collected during full-term C-sections from the Obstetrics and Gynaecology faculty, Heinrich Heine University Düsseldorf, Germany, with patient consent as well as institutional ethical approval and kept at 4°C until processed. In general, the time between collection and processing was kept as short as possible to minimize cell death. First, AF was washed with PBS (Gibco; Thermo Fisher Scientific, Darmstadt, Germany) and centrifuged at 300 ×g for 5 min. The supernatant was discarded, and the pellet washed again with PBS and was dissolved in Ammonium chloride (University Hospital Düsseldorf; Pharmacy) to lyse the remaining erythrocytes. Thereafter, the cell solution was incubated at 4°C for 20 min and centrifuged again. This procedure was repeated until the pellet had a clear colour. Afterwards, the cells were cultured in Chang C Medium (Irvine Scientific, CA, USA) containing 88% *α*MEM (Minimum Essential Medium Eagle Alpha Modification; Sigma) with 10% FBS, 1% GlutaMAX, 1% penicillin/streptomycin (all Gibco), 10% Chang B Basal Medium, and 2% Chang C supplement (Irvine Scientific) at 37°C and 5% CO_2_. Once attached, the cells were visible after 4–7 days and the medium was changed. Upon attainment of 90% confluency, the cells were detached using TrypLE Express (Thermo Fisher Scientific) and seeded into other plate formats or frozen.

### 2.2. Flow Cytometric Analysis of Amniotic Fluid Cells

The immunophenotyping of three independent AF preparations was done using the human MSC phenotyping kit (Miltenyi Biotec GmbH, Bergisch Gladbach, Germany) was done according to manufacturer's instructions. After harvesting, 2 × 10^5^ AF-MSCs were transferred into two test tubes. 2 ml PBS was added to each tube and centrifuged at 300 ×g for 5 min. The supernatant was discarded and the pellet resuspended in 100 *μ*l PBS within the tubes. In one of the tubes, 0.5 *μ*l of the MSC phenotyping cocktail and the other tube 0.5 *μ*l of the isotype control cocktail were added and vortexed. The MSC phenotyping cocktail contained fluorochrome-conjugated monoclonal antibodies CD14-PerCP, CD20-PerCP, CD34-PerCP, CD45-PerCP, CD73-APC, CD90-FITC, and CD105-PE. The isotype phenotyping cocktail contained fluorochrome-conjugated antibodies that should not specifically detect human antigens and was therefore used as a negative control. The tubes were incubated at 4°C for 10 min in the dark. To washout nonbinding antibodies, 1 ml PBS was added and centrifugation at 300 ×g for 5 min was performed. Afterwards, the cell pellet was fixed using 4% paraformaldehyde (PFA; Polysciences Inc., PA, USA). For flow cytometric analysis of the AF-MSCs for pluripotency-associated markers, TRA-1-60, TRA-1-81, and SSEA4 dye-coupled antibodies were used (anti-TRA-1-60-PE, human (clone: REA157), number 130-100-347; anti-TRA-1-81-PE, human (clone: REA246), number 130-101-410, and anti-SSEA-4-PE, human (clone: REA101), number 130-098-369; Miltenyi Biotec GmbH). The staining procedure was carried out as described above.

The cells were stored at 4°C in the dark until flow cytometric analysis via BD FACSCanto (BD Biosciences, Heidelberg, Germany) and CyAn ADP (Beckman Coulter, CA, USA) was done. Histograms were created using the FCSalyzer software version 0.9.3.

### 2.3. Immunofluorescence Staining

To analyse the cells for specific markers, AF-MSCs were cultured in 12- or 24-well plates. At 60–80% confluency, the cells were washed and subsequently fixed using 4% PFA for 15 min at room temperature (RT) on a rocking platform. The fixed cells were treated with 1% Triton X-100 (Carl Roth GmbH & Co. KG, Karlsruhe, Germany) for 5 min and blocking buffer was added to the cells. For intracellular staining, this buffer contained 10% normal goat serum (NGS; Sigma), 0.5% Triton X-100, 1% BSA (Sigma), and 0.05% Tween 20 (Sigma), all dissolved in PBS. If extracellular structures were to be stained, Triton and Tween were omitted. After blocking for 2 h at RT, the first antibodies OCT-4A (C30A3) rabbit mAb number 2840, SSEA4 (MC813) mouse mAb number 4755, E-cadherin (24E10) rabbit mAb number 3195, vimentin (5G3F10) mouse mAb number 3390, TRA-1-60 mouse mAb number 4746, TRA-1-81 mouse mAb number 4745 (Cell Signalling Technology, MA, USA), CD133 PA2049 (Boster Bio, PA, USA), and c-Kit (H-300) rabbit polyclonal IgG (Tebu Bio, Offenbach, Germany) were diluted in blocking buffer/PBS and added to the cells with an incubation time of 1 h at RT. After washing for three times with 0.05% Tween 20 in PBS, the appropriate secondary Cy3- or Alexa Fluor 488-labelled antibodies (Thermo Fisher Scientific) and Hoechst 33258 dye (Sigma-Aldrich Chemie GmbH, Taufkirchen, Germany, 1 : 5000 in blocking buffer) were applied for visualization of the primary antibodies and cell nuclei, respectively. Images were taken with a fluorescence microscope (LSM700; Zeiss, Oberkochen, Germany).

### 2.4. In Vitro Differentiation into Adipogenic, Chondrogenic, and Osteogenic Lineage

The differentiation of the AF-MSCs from passages 5 to 6 was carried out using the StemPro Adipogenesis differentiation Kit, StemPro Chondrogenesis differentiation Kit, and StemPro Osteogenesis differentiation Kit (Gibco, Life Technologies, CA, USA). The differentiation media were formulated by mixing 90 ml of the respective basal media with 10 ml of their corresponding supplements and 1.1 ml of penicillin/streptomycin. At 60–70% confluency, cultivation of the cells in the differentiation media or Chang C media (control wells) was initiated. The medium was replaced every 2-3 days for three weeks. After this period, the medium was removed, and the cells were washed with PBS and fixed with 4% PFA for 30 min at RT on a rocking platform. Subsequently, the cells were stained for distinct developed structures.

### 2.5. Oil Red O Staining for Adipocytes

Fixed cells were washed with 50% ethanol and then 0.2% Oil Red O working solution was added to the wells and incubated for 30 min at RT on a rocking platform. This solution stained the developed fat vacuoles. The 0.2% Oil Red O working solution was prepared by diluting the 0.5% Oil Red O stock solution (Sigma) with distilled water and filtering it. After washing twice with 50% ethanol and at least 3 times with distilled water until all the excess Oil Red O solution was removed, cells were kept in PBS and images were taken with a light microscope.

### 2.6. Alcian Blue Staining of Chondrocytes

After fixation, cells should be stained with alcian blue which turns sulfated proteoglycans deposits in chondrocytes visibly blue. Cells were first washed with PBS and 1% alcian blue 8GX (Sigma) solution, prepared in 0.1 N hydrochloric acid (HCl), was added. The cells were stained for 30 min at RT on a rocking platform. Afterwards, the cells were washed three times with 0.1 N HCl solution and with distilled water until the alcian blue solution was completely removed. Cells were then kept in PBS for microscopic imaging.

### 2.7. Alizarin Red S Staining for Osteoblasts

Alizarin red S (Sigma) which specifically stains developed calcium deposits was used to stain the cells after osteogenic differentiation. Cells were washed after fixation with PBS, and 2% alizarin red S solution in distilled water was added. After 30 min incubation at RT on a rocking platform, the cells were washed with distilled water and then with PBS to remove the remaining dye. For light microscopic analysis, the cells were kept in PBS.

### 2.8. Secretome Analysis

For the detection of cytokines secreted by the AF-MSCs, the Proteome Profiler Human Cytokine Array Panel A (R&D Systems, MA, USA) was performed according to the manufacturer's instructions. Initially, 1.5 ml of conditioned medium (pooled equal volumes from the three independent AF-MSC samples used for this study) was used. The array was evaluated by detection of the emitted chemiluminescence. The pixel density of each spotted cytokine was analysed using the software ImageJ. All spots on the membrane including reference and negative control spots were measured separately. Correlation variations and *p* values were calculated based on the pixel density. The pixel density value of 50 was set as the threshold.

### 2.9. RNA Isolation

After incubation with TRIzol (Thermo Fisher) for 5 min at RT on a rocking platform, the cells were detached and frozen within this solution at −80°C. The RNA was then isolated by using the Direct-zol RNA MiniPrep Kit (Zymo Research, CA, USA) which already contains DNase. The resulting RNA was dissolved in RNA/DNAse free water and analysed using the NanoDrop 2000 (Thermo Fisher) spectrophotometer.

### 2.10. Transcriptome Analysis

Microarray experiments were performed on the PrimeView Human Gene Expression Array (Affymetrix, Thermo Fisher Scientific) for two samples of AF-MSCs (AF-MSC1, AF-MSC2), foetal bone marrow-derived MSCs (fMSC), and embryonic stem cells (H1, H9) as well as human foreskin fibroblast-derived induced pluripotent stem cells (iPSCs) and are provided online at the National Center of Biotechnology Information (NCBI) Gene Expression Omnibus (https://www.ncbi.nlm.nih.gov/geo/query/acc.cgi?acc=GSE100448). The unnormalized bead summary data was further processed via the R/Bioconductor [30] environment using the package affy (http://bioconductor.org/packages/release/bioc/html/affy.html) [31]. The obtained data was background-corrected, transformed to a logarithmic scale (to the base 2), and normalized by employing the Robust Multiarray Average method. Heatmaps and cluster analysis were generated using the heatmap.2 function from the gplots package, and the correlation coefficients were measured using Pearson correlation as similarity measure (http://CRAN.R-project.org/package=gplots).

### 2.11. Gene Ontology, KEGG Pathway, and STRING Network Analysis

After transcriptome analysis gene ontology terms and associated KEGG pathways [32] for the different gene sets were generated using the DAVID tool (https://david.ncifcrf.gov/) [33], the STRING network tool was used for network cluster analysis (https://string-db.org/) [34].

## 3. Results

### 3.1. Isolation and Culture of C-Section-Derived AF-MSCs

**Figure 1:**
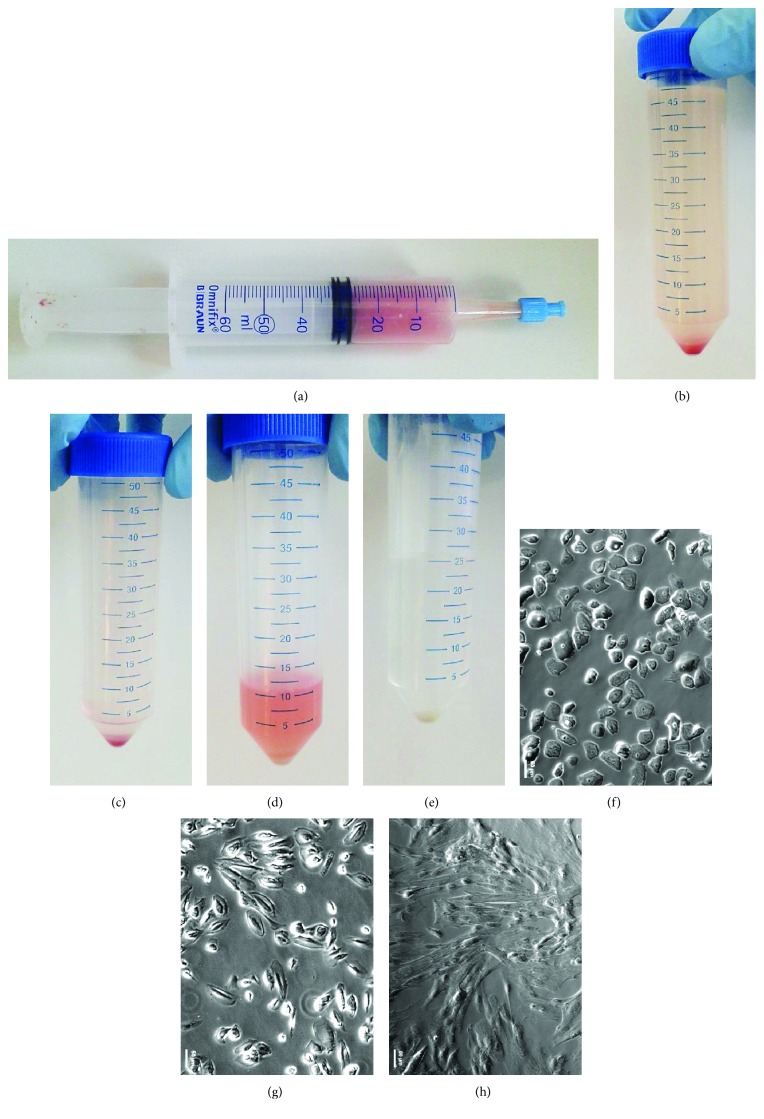
Amniotic fluid cell preparation. Amniotic fluid was collected during Caesarean sections using a 60 ml syringe (a). The AF was washed with PBS and centrifuged resulting in a reddish pellet (b, c). The red colour indicated the presence of erythrocytes which were then lysed using ammonium chloride at 4°C. The resulting white cell pellet (d, e) was transferred to cell culture vessels showing high level of heterogeneity (f). After, prolonged *in vitro* culture and passaging heterogeneous cell morphology (g) became uniform showing spindle-shaped fibroblast-like morphology (h).

During C-sections at full-term gestation, AF was collected using a syringe (Figure 1(a)) and transferred into 50 ml tubes. The red colour of the fluid indicates the presence of erythrocytes. The AF was washed twice with PBS (Figures 1(b) and 1(c)) then the remaining erythrocytes were lysed by resuspending the cell pellet in ammonium chloride (Figure 1(d)). After additional washing, the pellet had a whitish colour indicating successful removal of the remaining blood cells (Figure 1(e)). Microscopic analysis directly after the purification displayed a heterogeneous mixture of different cell types (Figure 1(f)). First, attached cells were visible after 4 to 7 days. After passaging them twice, the heterogeneous morphology of the cells (Figure 1(g)) became more homogeneous with spindle-shaped fibroblast-like forms (Figure 1(h)). Cells were cultured until they all showed a homogeneous MSC morphology and then used for further experiments.

### 3.2. In Vitro Differentiation Capacity and Cell Surface Marker Expression

**Figure 2:**
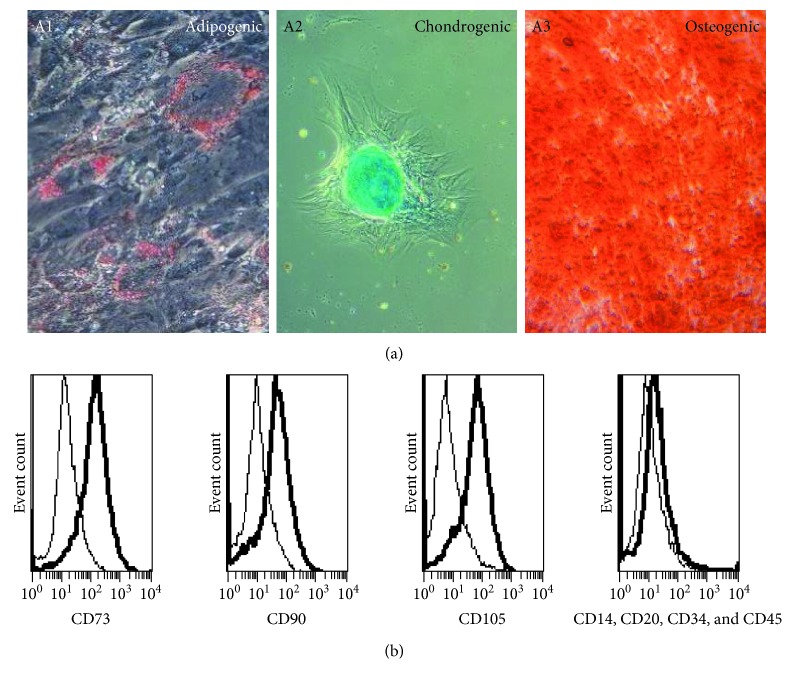
*In vitro* differentiation potential and immunophenotype of AF-MSCs. Multilineage differentiation potential of AF-MSCs was investigated by applying adipocyte, chondrocyte, and osteoblast differentiation media to the cells for 3 weeks. Staining of Oil Red O showed successful differentiation into adipogenic lineage with developed fat vacuoles surrounding the cell nucleus (A1). Chondrogenic differentiation was shown to be present by alcian blue staining of cell aggregates containing proteoglycan (A2). Osteoblast formation by AF-MSCs was indicated by alizarin red S staining of calcium deposits (A3). Flow cytometry was used for the analysis of cell surface marker expression. Histograms showed that MSC markers CD73, CD90, and CD105 were detected as cell surface proteins on AF-MSCs preparation derived from C-sections whereas hematopoietic marker expressions CD14, CD20, CD34, and CD45 were low (bold lines) (b). Antibody isotype controls are represented by thin lines.

To investigate their multipotent differentiation capacity, AF-MSCs from three independent preparations were challenged to differentiate into adipogenic, chondrogenic, and osteogenic directions by employing distinct differentiation media for 3 weeks. Successful differentiation into adipocytes was observed by staining of emerging fat droplets with Oil Red O solution (Figure 2(a), A1). The fat vacuoles surrounded the cell nuclei. During chondrogenic differentiation, the cells aggregated and alcian blue staining showed the presence of emerged proteoglycans within the developed cell clusters of chondrocytes (Figure 2(a), A2) and osteogenic lineage differentiation was shown by alizarin red S staining of developed calcium deposits (Figure 2(a), A3). The visual mineralization of the cells started after the first week. Cells of the control wells remained fibroblast-like. Cells from all preparations showed a higher propensity to differentiate into the osteogenic lineage than into the other two investigated lineages as evidenced by differentiated areas within the cell culture dish of about 90%.

### 3.3. Flow Cytometric Analysis for Cell Surface Marker Expression

**Figure 3:**
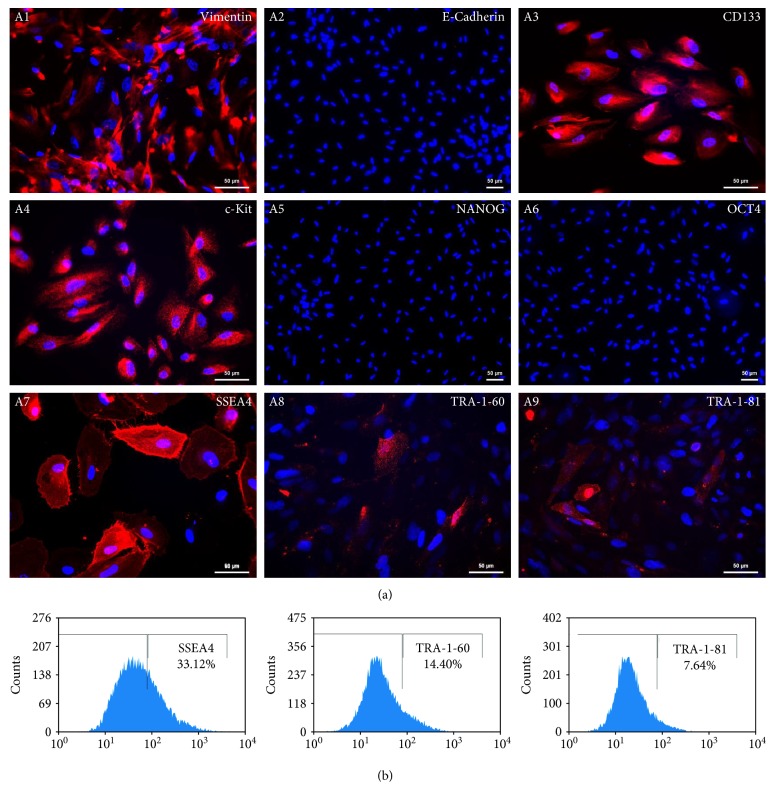
Protein expression analysis of AF-MSCs. (a) By immunofluorescent staining, AF-MSCs were found to express vimentin (A1), CD133 (A3), c-Kit (A4), SSEA4 (A7), TRA-1-60 (A8), and TRA-1-81 (A9) and by parallel absence of E-cadherin (A2), NANOG (A5), and OCT4 (A6). Cell nuclei were stained using Hoechst. (b) Flow cytometric analysis confirmed cell surface expression of SSEA4 (B1), TRA 1-60 (B2), and TRA-1-81 (B3).

To analyse the cell surface marker presence on the AF-MSCs, the human MSC phenotyping kit (Miltenyi Biotec GmbH, Bergisch Gladbach, Germany) was used which contained antibodies against MSC-related markers CD73, CD90, and CD105 separately and antibodies against haematopoietic markers CD14, CD20, CD34, and CD45 in a combined cocktail. After staining, the cells were analysed using a flow cytometer. Within the three independent AF-MSC preparations, the presence of CD73, CD90, and CD105 positive cells was up to 90%. As expected, all cell preparations were devoid of the haematopoietic markers CD14, CD20, CD34, and CD45 (Figure 2(b)). Furthermore, AF-MSCs were analysed for the expression of pluripotency-associated cell surface markers. A subpopulation of approximately 33% of the cells was positive for SSEA4 whereas 14% of the cells was positive for TRA-1-60 and 8% was positive for TRA-1-81 (Figure 3(b), B1, B2, and B3).

### 3.4. Immunofluorescent-Based Analysis of Stem Cell Marker Expression in AF-MSCs

AF-MSCs had a spindle-shaped morphology and expressed the type III intermediate filament vimentin (Figure 3(a), A1) which is expressed by mesenchymal cells and widely used as a mesenchymal indicator [35]. In parallel, these cells were negative for E-cadherin (Figure 3(a), A2) as a marker for epithelial cells. The expression of CD133/prominin-1 (Figure 3(a), A3), a marker for multipotent progenitor cells including MSCs [36, 37], was detected. The populations we isolated did not express OCT4 or NANOG (Figure 3(a), A5 and A6). However, the AF-MSCs expressed c-Kit, SSEA4, TRA-1-60, and TRA-1-81 (Figure 3(a), A4, A7, A8, and A9). The percentage of cells positive for the investigated markers was consistent with the flow cytometric data.

### 3.5. Secretome Analysis

**Figure 4:**
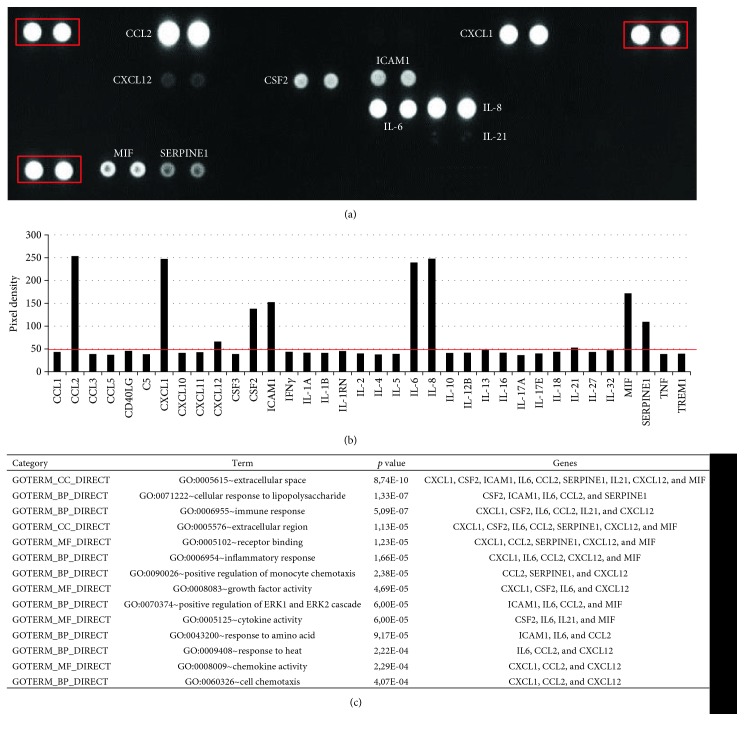
Secretome analysis of AF-MSCs. The cytokines CCL2, CXCL1, CXCL12, CSF2, ICAM, IL-6, IL-8, IL-21, MIF, and SERPINE1 were detected by protein arrays in cell culture supernatant from AF-MSCs. A membrane with spotted antibodies was used for detection. The three red-boxed spot pairs in the corners represent protein array quality controls (a). Densitometric analysis revealed specific pixel densities; the pixel density of 50 represents the threshold (red line) (b). Gene ontology analysis of secreted cytokines revealed the shown top 14 results with *p* values below 0.0005 (c).

The ability of AF-MSCs to secrete cytokines was investigated employing a cytokine array. To achieve this, cell culture supernatants from three distinct AF-MSC preparations were pooled and analysed using the cytokine array. This revealed the presence of chemokine (C-C motif), ligand 2 (CCL2; MCP-1), C-X-C motif chemokine 1 (CXCL1; GRO*α*), CXCL12 (SDF-1), colony stimulating factor 2 (CSF2; GM-CSF), intercellular adhesion molecule 1 (ICAM1; CD54), interleukin-6 (IL-6), IL-8, IL-21, macrophage migration inhibitory factor (MIF), and plasminogen activator inhibitor-1 (PAI-1; SERPINE1) at distinct levels which were above background expression (Figures 4(a) and 4(b)). CCL2, CXCL1, IL-6, and IL-8 were the highest secreted cytokines. Average levels of secretion were found for CSF2, ICAM1, MIF, and SERPINE (PAI-1) whereas CXCL12 and IL-21 were expressed at the lowest levels. Gene ontology term analysis of the secreted cytokines revealed terms associated with immune modulatory properties such as “immune response,” “chemotaxis,” and “inflammatory response” (Figure 4(c)).

### 3.6. Overlapping, Distinct Gene Expression, Associated Gene Ontologies, and Pathways

**Figure 5:**
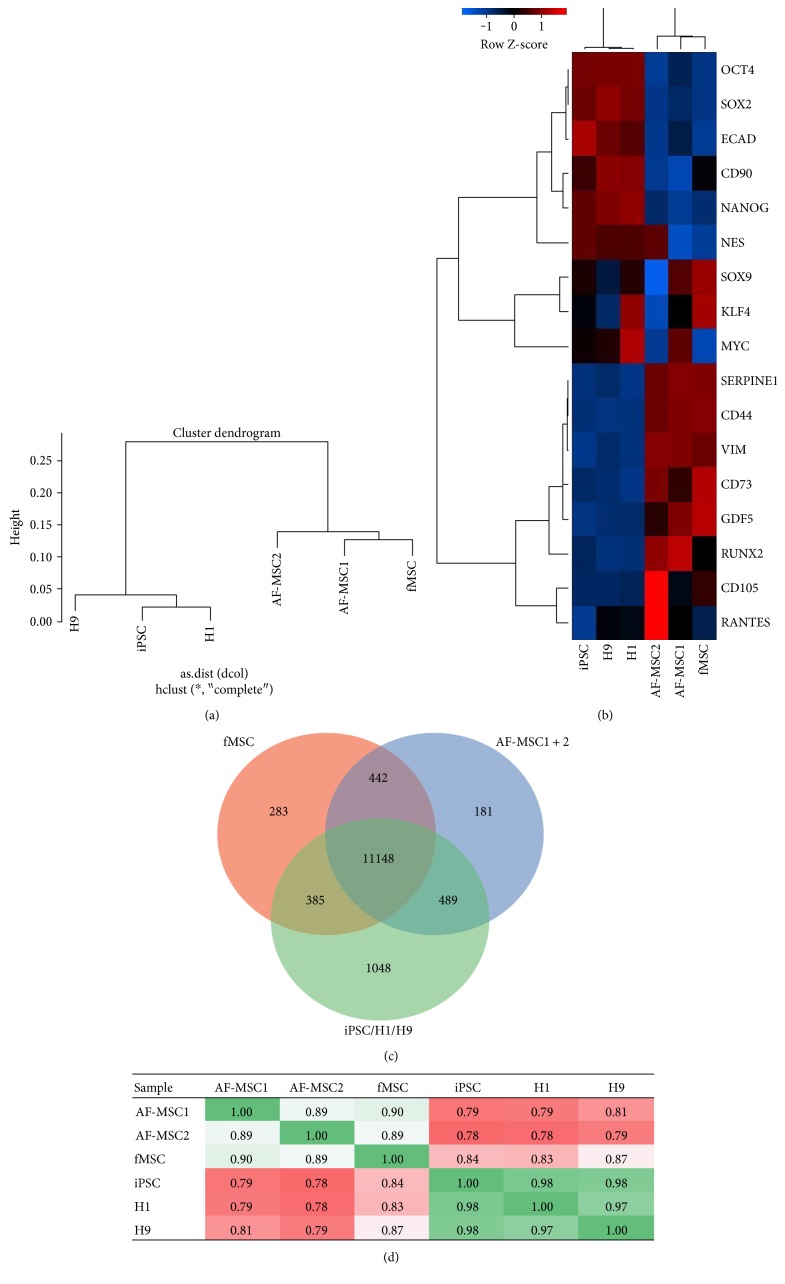
Overlapping and distinct gene expression in AF-MSCs. Dendrogram resulting from hierarchical clustering (a) of global gene expression profiles of AF-MSCs, fMSCs, and established pluripotent stem cells (H1, H9, and iPSCs). Transcriptomes of AF-MSC1 and AF-MSC2 cluster with fMSC while those of the H1, H9, and iPSCs cluster separately. The heatmap of 17 commonly up- or downregulated genes (b) shows similar gene expression of AF-MSCs and fMSCs. Venn diagram analysis revealed shared gene expression between fMSC, AF-MSCs, and pluripotent stem cells (c). Pearson's correlation coefficient was calculated based on the transcription data (d). Each replicate was pairwise compared with each other replicate. A value of 1 indicates perfect linear correlation while a value of 0 implies no correlation. Pearson correlation analysis of transcriptome data revealed a high correlation (green) of both AF-MSC1 and AF-MSC2 with fMSCs but low correlation (red) with pluripotent cells.

Hierarchical clustering (Figure 5(a)) based on the transcriptome profiles of AF-MSCs, fMSCs, and pluripotent stem cells (H1, H9, and iPSCs) revealed a closer relationship of AF-MSC1 and AF-MSC2 to native fMSCs than to pluripotent stem cells. The heatmap derived from the transcriptome data (Figure 5(b)) shows that the cells from both AF preparations are closer to fMSCs. The heatmap consists of 17 genes commonly up- and downregulated between AF-MSCs, fMSCs, and pluripotent cells. Genes which were expressed predominantly in AF-MSCs and fMSCs were vimentin *(VIM)*, *CD44*, *CD73*, *CD105*, and *SERPINE1* as well as osteogenic markers runt-related transcription factor 2 (*RUNX2*) and growth/differentiation factor 5 (*GDF5*). In contrast to this, AF-MSCs and fMSCs were devoid of *E-Cadherin* and pluripotency markers *OCT4*, *SOX2*, and *NANOG*. Venn diagram analysis (Figure 5(c)) revealed an overlap of 11,148 genes among all cell types. Interestingly, AF-MSCs shared more genes (489) with pluripotent stem cells than with fMSCs (442). KEGG pathway analysis of genes shared between pluripotent stem cells and AF-MSCs showed the involvement of phosphatidylinositol pathway and Notch signalling (Supplementary Figure S1 available online at https://doi.org/10.1155/2017/5932706). However, AF-MSCs distinctly expressed 181 genes.

Pearson correlation analysis of the transcriptome data (Figure 5(d)) revealed a high correlation (0.89 and 0.90) between AF-MSC1 and AF-MSC2 and fMSCs but low correlation (0.78–0.81) with the pluripotent cells. The significant KEGG pathways as well as gene ontology terms related to the shared genes between AF samples and fMSCs were related to immune function, skeletal development, and TGF*β*-signalling (Figure S2).

### 3.7. AF-MSC-Specific Gene Expression Analysis

**Figure 6:**
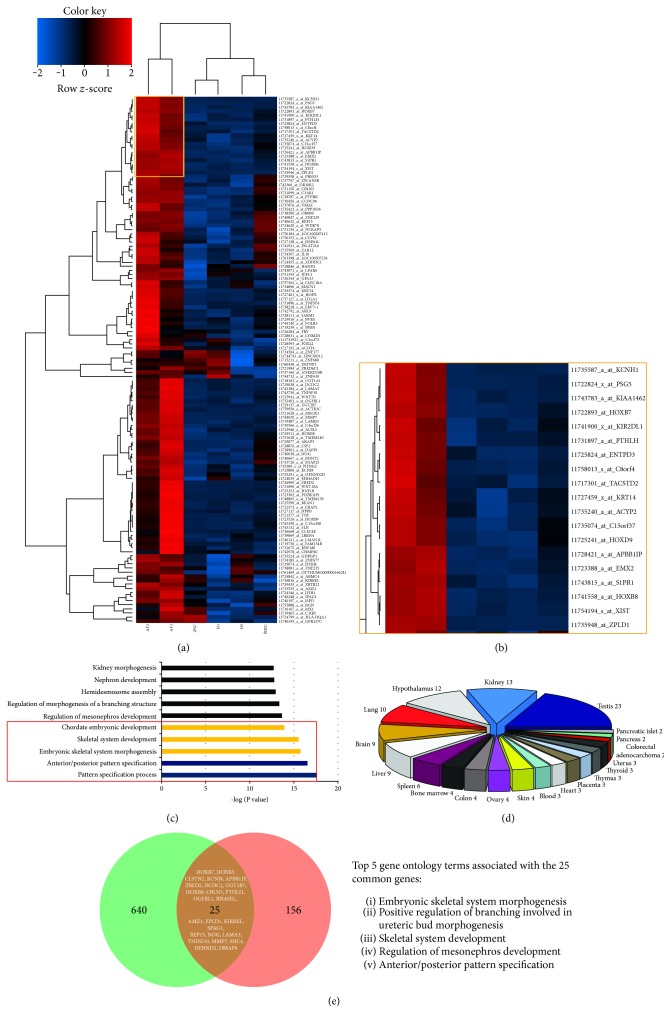
AF-MSC-specific genes. (a) Heatmap and clustering of the 181 genes exclusive for AF-MSCs (see Figure 5(c)). Zoom in on one cluster of the heatmap (b) showed possible AF-MSC markers (yellow box). Gene ontology (GO) analysis of the 181 AF-MSC exclusive genes using the DAVID tool for the GO terms associated with biological processes (c) with a maximum *p* value of 0.05. Significantly enriched GO terms for each category are shown with the −log of their *p* values. (d) Tissue distribution of the 181 exclusive AF-MSC-related genes. (e) Comparative analysis of 181 AF-MSC-specific genes and an already published data set of 665 AFSC-specific genes [11] via Venn diagram uncovered a common set of 25 genes.

A heatmap was derived using the 181 AF-MSC exclusive gene set (Figure 6(a)). One subset of these genes could be used to identify possible AF-MSC marker genes (Figure 6(b)) including *PSG5*, *C4orf26*, *C8orf4*, *EVR-3*, *EMX-2*, and *C15orf37*. Furthermore, gene ontology analysis focusing on biological processes (Figure 6(c)) showed the involvement of genes associated with skeletal system development and patterning. Tissue-specific gene distribution analysis (Figure 6(d)) revealed the relationship between the 181 AF-MSC-specific genes and the different embryonic tissues. The most prominent tissues were the testis, kidney, and hypothalamus. The rest of the genes were distributed among the other organs. The AF-MSC-specific gene set (181 genes) was further compared to an already published transcriptome dataset of third-trimester AFSCs [11] and visualized with a Venn diagram (Figure 6(e)). 25 genes including *HOXB7*, *APBB1IP*, *HOXB8*, *PTHLH*, and *ZPLD1* were found to be expressed in common between these two gene sets. Referring to the associated gene ontology terms, these genes are mostly associated with embryonic skeletal system morphogenesis, positive regulation of branching involved in ureteric bud morphogenesis, skeletal system development, and regulation of mesonephros development as well as anterior/posterior pattern specification.

### 3.8. Network Analysis of 181 AF-MSC Exclusive Genes

**Figure 7:**
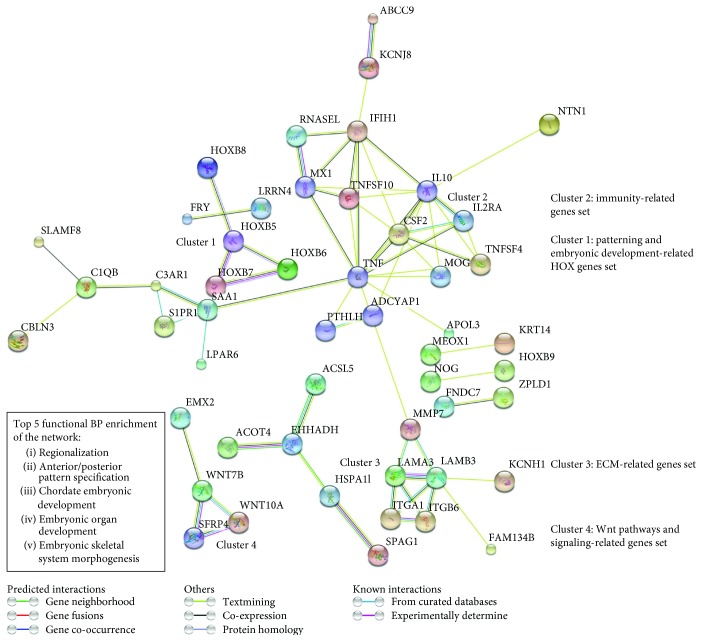
Network analysis of the 181 AF-MSC-specific genes. STRING network analysis revealed 4 distinct clusters: cluster 1 represents the genes involved in patterning and embryonic development which mainly involves the HOX genes. Cluster 2 contains genes related to immunity (e.g., TNF and IL-10). Extracellular matrix- (ECM-) related genes are found in cluster 3, and cluster 4 shows genes related to WNT signalling.

The network analysis of the 181 AF-MSC-specific genes was done using the STRING tool and predicted 4 different clusters (Figure 7): cluster 1 displayed the patterning and embryonic development related HOX genes such as the homeobox B7 (*HOXB7*), cluster 2 contained the immunity-related gene (e.g., *CSF2*), and cluster 3 included the extracellular matrix- (ECM-) related gene set (e.g., laminin subunit alpha 3 (*LAMA 3*)) whereas cluster 4 showed the WNT pathway and signalling-related gene set which includes *WNT10A*. The network analysis also revealed functional biological process (BP) enrichment of regionalization, anterior/posterior pattern specification, chordate embryonic development, embryonic organ development, and embryonic skeletal system morphogenesis.

## 4. Discussion

In comparison to our results, it was shown that MSCs derived from the bone marrow attached to the cell culture dish within three days after plating whereas umbilical cord and adipose tissue-derived MSCs attached within the first 24 h [38]. The prolonged attachment time of the AF-MSCs could be explained by the change of environment (microenvironment of amnion with distinct chemicals), the high heterogeneity of the cells, and a lower prevalence of stem cells within at term amniotic fluid when compared to the first and second-trimester amniotic fluid. In addition to the fibroblast-like cells, other cell morphologies were present in the AF preparation but these diluted out with time and increasing passage numbers (Figure 1). The remaining cells were of mesenchymal morphology and expressed vimentin and were devoid of E-cadherin (Figure 2(a), A1 and A2). Wolfrum et al. reported epithelial-like morphologies in senescent AFSCs [11]. Hoehn et al. similarly recognized different populations in second-trimester AF that either possessed fibroblast-like or epithelial-like morphologies [39, 40]. Comparable proliferative capabilities of the populations, long-lasting ex vivo culture of the fibroblast-like cells which proliferated over 30 passages and the epithelial-like morphologies have also been observed. Nevertheless, most of the studies obtained AFSCs from amniocentesis. This procedure has restricted access to the fluid with a certain level of risk to the foetus and mother [41]. The collection of AF from full-term pregnancies or during deliveries as done in the present study could be a possible alternative option with higher prevalence of healthy diploid foetuses as compared to first- and second-trimester-derived AF.

The exhibited multilineage differentiation potential into bone, fat, and cartilage cells of the C-section-derived AF-MSCs (Figure 2(a), A1, A2, and A3) was also previously described for AFSCs derived from amniocentesis. The chondrogenic differentiation potential of AF-MSCs derived from amniocentesis has been reported [42] and especially osteogenic differentiation potential and further use in bone defect models underlines the potency of these cells to build up osteoblasts [43] and thus could be used for future bone related therapies.

The AF-MSCs obtained during C-sections showed the typical MSC cell surface marker expression of CD73, CD90, and CD105 by parallel absence of the haematopoietic markers CD14, CD20, CD34, and CD45 (Figure 2(b)) as described by the ISCT [24]. However, as shown in previous studies, different individuals and origins of MSCs, respectively, can lead to altered cell surface maker expressions [23].

The transcription factor OCT4 in association with NANOG and SOX2 has been shown to be the key driver of pluripotency [29]. However, the majority of AFSC studies published so far have focused on only expression but not function.

In contrast to other studies describing OCT4 and NANOG expression in third-trimester AF-derived cells, our analysis revealed these markers to be negative for caesarean-derived AF-MSCs [27, 28, 41]. In our case, we identified singular cytoplasmic OCT4 positive-expressing cells at passages 1-2, but after a few more passaging, these cells were diluted out (data not shown). This variability could be due to the number of passages, different protocols, culture methods, and media used.

It was previously shown that a subpopulation of AFSCs from the first and second trimester expresses the pluripotency markers OCT4, NANOG, SOX2, c-MYC, and KLF4 [6, 7, 10] thus indicating that first and second-trimester AFSCs are multipotent and express pluripotency-associated markers. Although, Prusa et al. claimed OCT4 expression by 5 out of 11 independent AF-MSCs (no passage numbers stated) as indicated by real-time PCR. Additionally, they only showed a single cell of their AF preparations being positive for OCT4. Furthermore, the functionality of this OCT4 positivity was not addressed [6].

However, first-trimester c-Kit-positive AFSCs were converted to a pluripotent state by supplementation with valproic acid in pluripotency supporting media and matrix. [10]. While valproic acid could induce pluripotency, these cells were distinct from hESCs as evidenced by microarray analysis [9]. Additional results [7] further support our observation that developmental potential of AFSCs decreases with gestation time. Also shown in our current study, transcriptome cluster analysis revealed clear separation between AFSCs and hESCs [7]. Thus it can be concluded that subpopulations of early term AFSCs are more susceptible for reprogramming events but are not pluripotent.

In this study, we have demonstrated the expression of SSEA4, an early embryonic glycolipid antigen by immunofluorescent and flow cytometric analysis (Figures 3(a), A7 and 3(b), B1). This protein does not play a critical role in maintaining pluripotency and has also been shown to be expressed in adult BM-MSCs [44, 45]. Furthermore, the cells were found to be c-Kit positive (Figure 3(a), A4), which is essential for the maintenance and differentiation of hematopoietic stem cells and multipotent progenitor cells [46]. It has been reported that c-Kit-expressing cells show a subpopulation of MSCs derived from adipose tissue that possess a higher telomerase expression and differentiation potential [47]. Moreover, a subpopulation of the AF-MSCs from C-sections also expresses TRA-1-60 and TRA-1-81 as shown by immunofluorescent-based stainings as well as flow cytometric analysis (Figures 3(a), A8 and A9 and 3(b), B2 and B3). This relates to already existing studies of midtrimester AF preparations [48].

Chemokines and their correspondent receptors are important for attraction and homing of leukocytes to infections, injury, or inflammation sites [49]. MSCs express these receptors, and thus it has been shown that chemokines and growth factors are chemotactic for bone marrow-derived MSCs [50]. Due to their immune modulatory properties, MSCs are widely used in clinical application in graft versus host disease [51]. In accordance with our secretome data (Figure 3) revealing the release of at least 9 distinct cytokines from AF-MSCs, Mirabella et al. analysed AFSC-conditioned media and identified the presence of known proangiogenic and antiangiogenic factors such as vascular endothelial growth factor (VEGF), CXCL12, IL-8, CCL2, two angiogenesis inhibitors interferon gamma (IFN*γ*), and CXCL10 and IP-10 as secreted proteins [13]. Besides angiogenesis, AFSCs also contribute to osteogenesis either directly or indirectly by secreting distinct cytokines [14].

The therapeutic potential of AF-MSCs and their secreted molecules in mice with acute hepatic failure has been analysed, and numerous proinflammatory mediators, regulating cytokines and growth factors in AF-MSC-conditioned media such as IL-10, IL-27, IL-17E, IL-12p70, IL-1*β*, and IL-1ra, were observed. Some tissue repair promoting factors, namely, SERPINE1, MCP-1, and SDF-1, were also identified [52]. Our results agree with the previous comparison of cytokines released from MSCs originating from the bone marrow, cord blood, and placenta. The pool of cytokines previously investigated was the same as that of our work. MIF, IL-8, SERPINE1, GRO*α*, and IL-6 were secreted by MSCs from all the investigated sources. Placental MSCs expressed ICAM-1 (CD54), and MCP-1 (CCL2) and bone marrow MSCs secreted MCP-1 (CCL2), and SDF-1 (CXCL12) in addition [53]. Other studies that investigated a larger pool of cytokines showed additional expression of RANTES, INF*γ*, IL-1*α*, TGF*β*, angiogenin, and oncostatin M [54]. The trophic factors released by AF-MSCs are and will be of great importance for future therapies.

Cluster dendrogram analysis clearly demonstrated that the transcriptomes of the two AF-MSC samples clustered together with the fMSC sample while the pluripotent iPSCs and ESCs (H1, H9) clustered in a separate group (Figure 5(a)). Both AF-MSC preparations acquired the expression profile of native foetal MSCs (Figures 5(b) and 5(d)). They were devoid of pluripotency-associated markers *OCT4*, *NANOG*, and *SOX2* but express the MSC markers *CD44*, *CD73*, *CD105*, and *vimentin*.

Recent studies investigating the gene expression pattern of AFSCs at different passages by illumina microarray detected 1970 differentially expressed genes and classified the expression profile into 9 distinct clusters. Genes with gradually increasing expression and higher passages included *CXCL12*, *CDH6*, and *FOLR3*. On the other hand, the important downregulated genes were *CCND2*, *K8*, *IGF2*, *BNP-B*, and *CRABPII* [55]. The Venn diagram of the analysed data sets in the present study showed a group of genes which are exclusively expressed by the AF-MSC samples (Figure 5(c)). From the heatmap of the 181 AF-MSC-specific genes (Figure 6(a)) identified by transcriptome analysis, a group of potential AF-MSC marker genes was extracted. This group contained *PSG5*, *C4orf26*, *C8orf4*, *EVR-3*, *EMX-2*, and *C15orf37* amongst others (Figure 6(b)), of which some such as *C8orf4* have not been characterized yet. Using these 181 genes, gene ontology analysis was conducted and most of the terms within the top 10 results of the biological processes were related to bone and skeletal development (Figure 6(c)). Global gene expression of AFSCs compared with AF-iPSCs and ESCs revealed genes related to self-renewal and pluripotency (1299 genes, e.g., *POU5F1*, *SOX2*, *NANOG*, and *microRNA-binding protein LIN28*) as well as AFSC-specific genes (665 genes, e.g., *OXTR*, *HHAT*, *RGS5*, *NF2*, *CD59*, *TNFSF10*, and *NT5E*) were identified in AFSCs [11]. The AF-MSC-specific genes from our study were further investigated using the STRING tool which built up 4 different clusters (Figure 7): patterning and embryonic development-related HOX genes, immunity-related genes, ECM-related genes, and a WNT pathway and signalling-related gene set which is in line with the KEGG pathway analysis (Figure S3). Compared to the previous identified 665 AFSC-specific genes, we could show an overlap of 25 genes (Figure 6(e)) which include *HOXB7*, *APBB1IP*, *HOXB8*, *PTHLH*, and *ZPLD1* which were also present within the highest expressed genes within our samples (Figure 6(b)). These genes are mainly involved in skeletal development (Figure 6(e) and Table S1). This subset of genes represents putative marker genes for AF-MSC selection procedures in the future.

## 5. Conclusion

In this study, we have demonstrated that a subpopulation of human AFSCs (AF-MSCs) isolated from AF collected during C-sections is indeed MSCs meeting the accepted criteria and definition [16]. In addition, we show that the transcriptomes of AF-MSCs are more similar to that of BM-MSCs (Pearson's correlation of 0.9) than to bona fide pluripotent stem cells (human embryonic stem cell lines H1 and H9 and a dermal fibroblast-derived iPSC line) even though they express well-known pluripotency-associated markers. We finally demonstrated their ability to secrete a plethora of cytokines and growth factors crucial for paracrine signalling. Overall, Caesarean section-derived amniotic fluid which in contrast to that obtained from amniocentesis is of no risk to the foetus and contain AF-MSCs with great potential for clinical applications.

## Supplementary Material

The information of supplementary materials are as follows: SUPPLEMENTARY FIGURE 1: AF-MSC and pluripotent cells shared genes top 12 KEGG pathways and significant gene ontologies for biological processes and cellular components. SUPPLEMENTARY FIGURE 2: AF-MSC and fMSCs shared genes top 19 KEGG pathways and significant gene ontologies for biological processes and cellular components. SUPPLEMENTARY FIGURE 3: 181 AF-MSC-specific genes significant KEGG pathways and top gene ontologies for cellular components. SUPPLEMENTARY TABLE S1: 25 AFSC-specific genes significant KEGG pathways and top gene ontologies for cellular components specific genes from the transcriptome analysis and a public available data set from Wolfrum et al. (2010).
