# Supplementary material for: Isolation and Molecular Characterization of Amniotic Fluid-Derived Mesenchymal Stem Cells Obtained from Caesarean Sections
Source: Stem Cells Int. 2017 Oct 31;2017:5932706. doi: 10.1155/2017/5932706 (PMC5684599; doi:10.1155/2017/5932706)

**Isolation and Molecular Characterization of Amniotic Fluid-derived Mesenchymal Stem Cells obtained from Caesarean sections**

**Authors:**

Lucas-Sebastian Spitzhorn^1*^, Md Shaifur Rahman^1*^, Laura Schwindt^1^, Huyen-Tran Ho^1^, Wasco Wruck^1^, Martina Bohndorf ^1^, Silke Wehrmeyer^1^, Audrey Ncube^1^, Ines Beyer^2^, Carsten Hagenbeck^2^, Percy Balan^2^, Tanja Fehm^2^ and James Adjaye^1#^

^* These authors contributed equally to this work^

**Supplementary Data**

Supplementary Figure 1: AF-MSC and pluripotent cells shared genes top 12 KEGG pathways and significant gene ontologies for biological processes and cellular components.

**
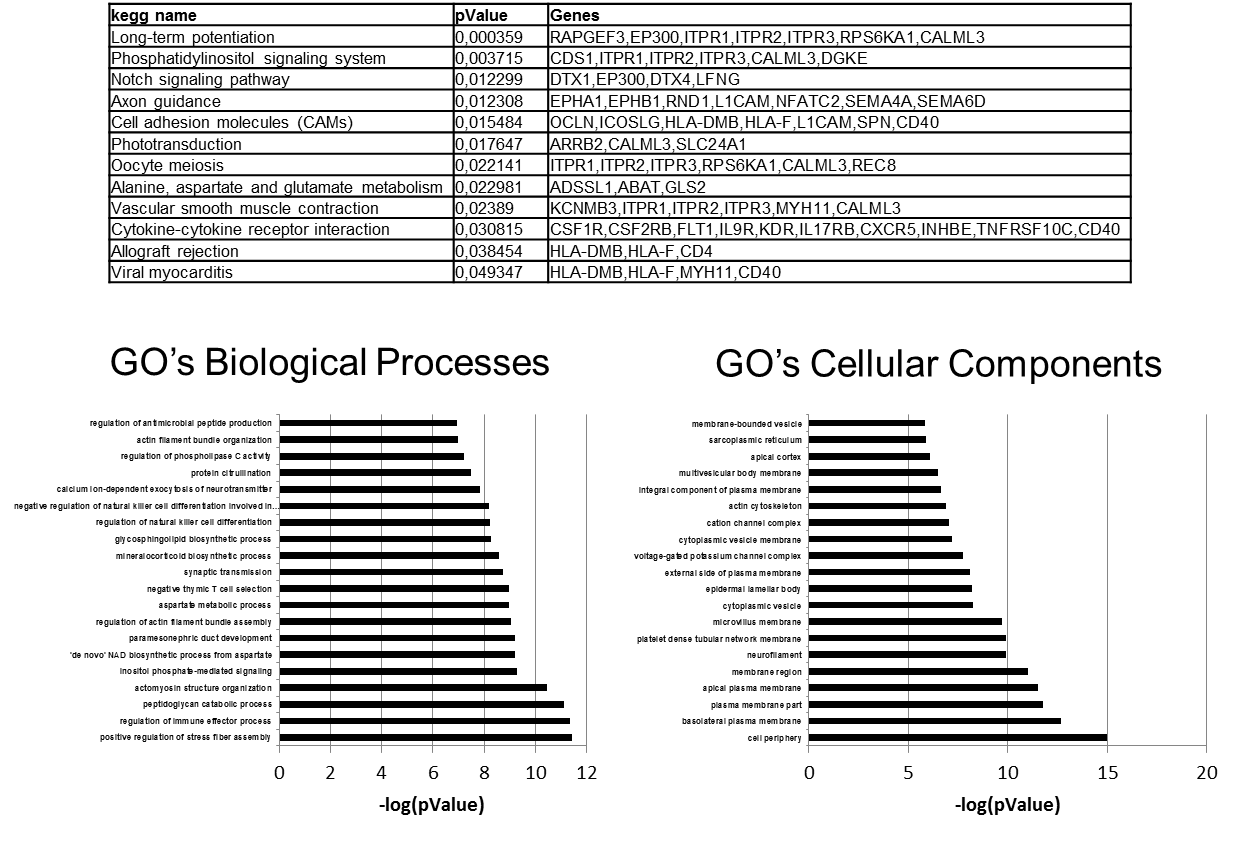
**

Supplementary Figure 2: AF-MSC and fMSCs shared genes top 19 KEGG pathways and significant gene ontologies for biological processes and cellular components.

**
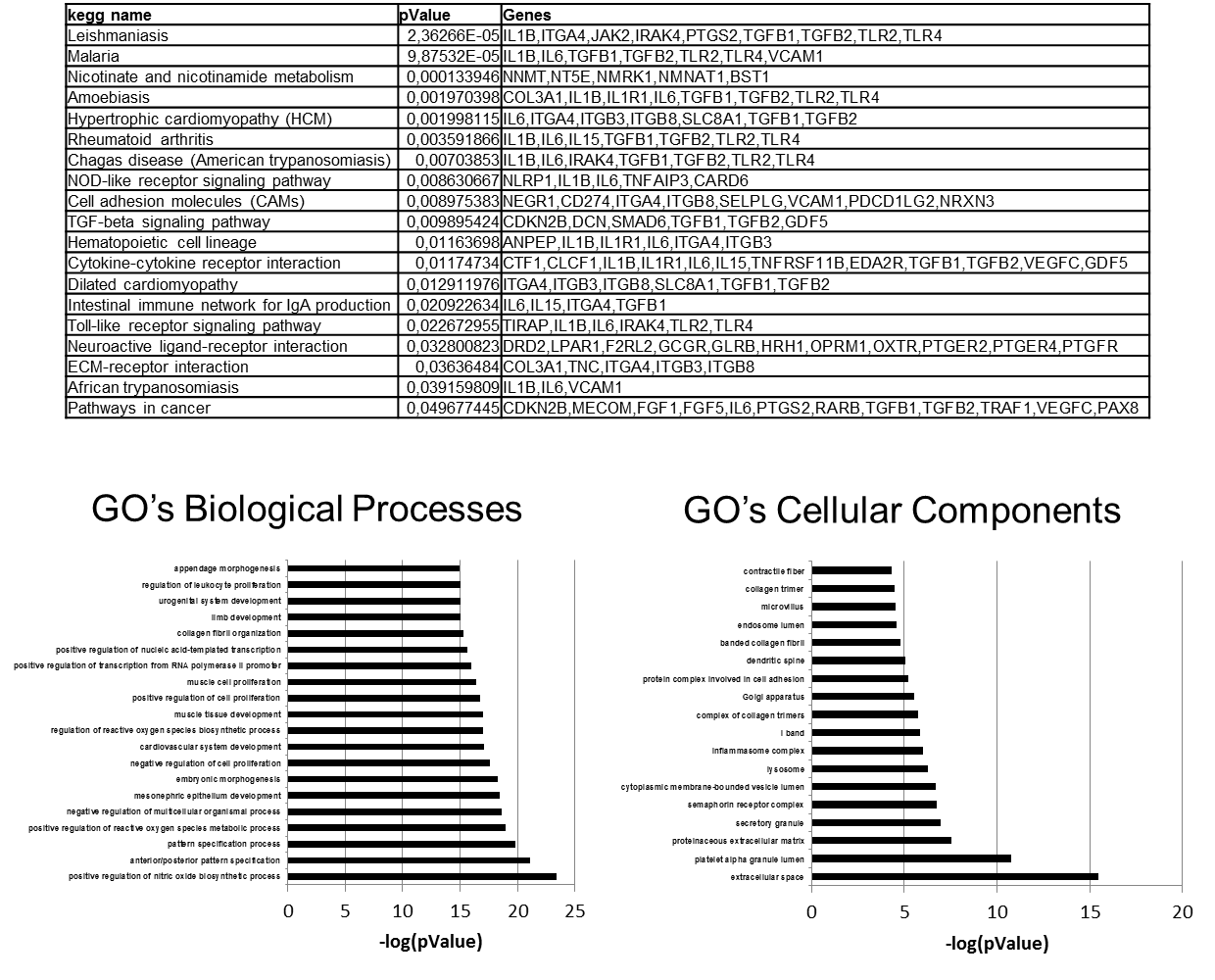
**

Supplementary Figure 3: 181 AF-MSC-specific genes significant KEGG pathways and top gene ontologies for cellular components.

**
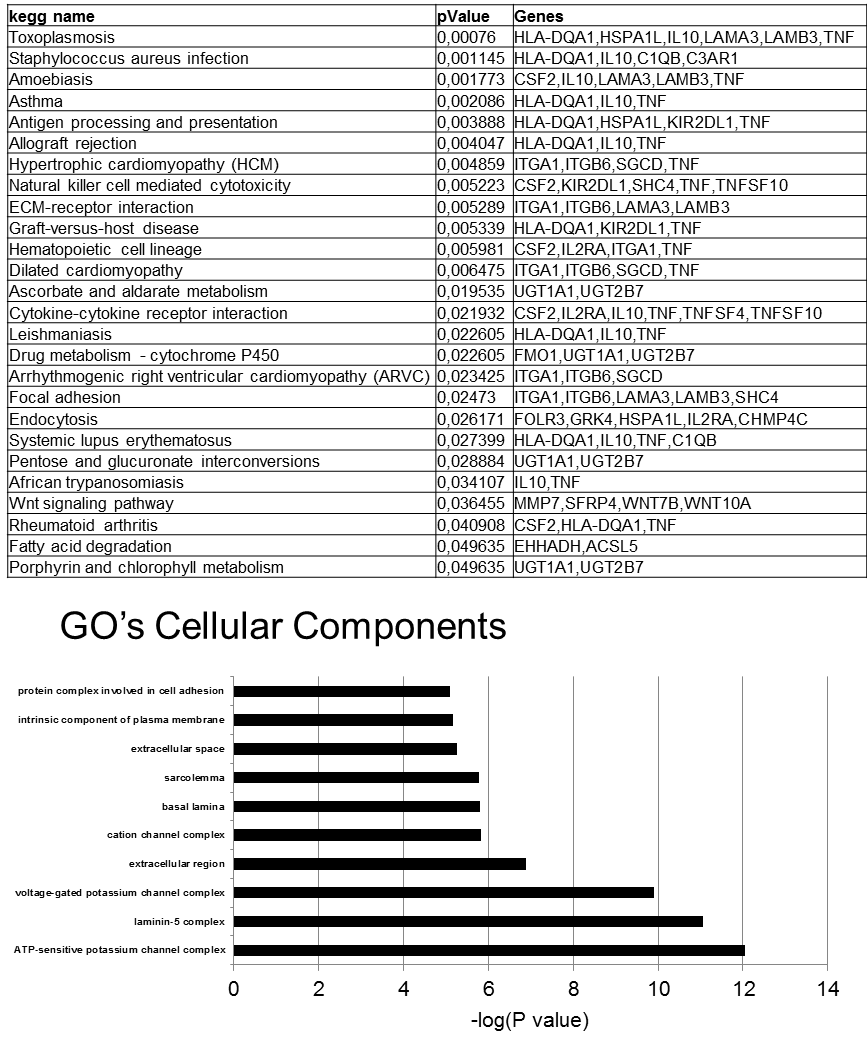
**

Supplementary Table S1: 25 AFSC-specific genes significant KEGG pathways and top gene ontologies for cellular components specific genes from the transcriptome analysis and a public available data set from Wolfrum et al. (2010).


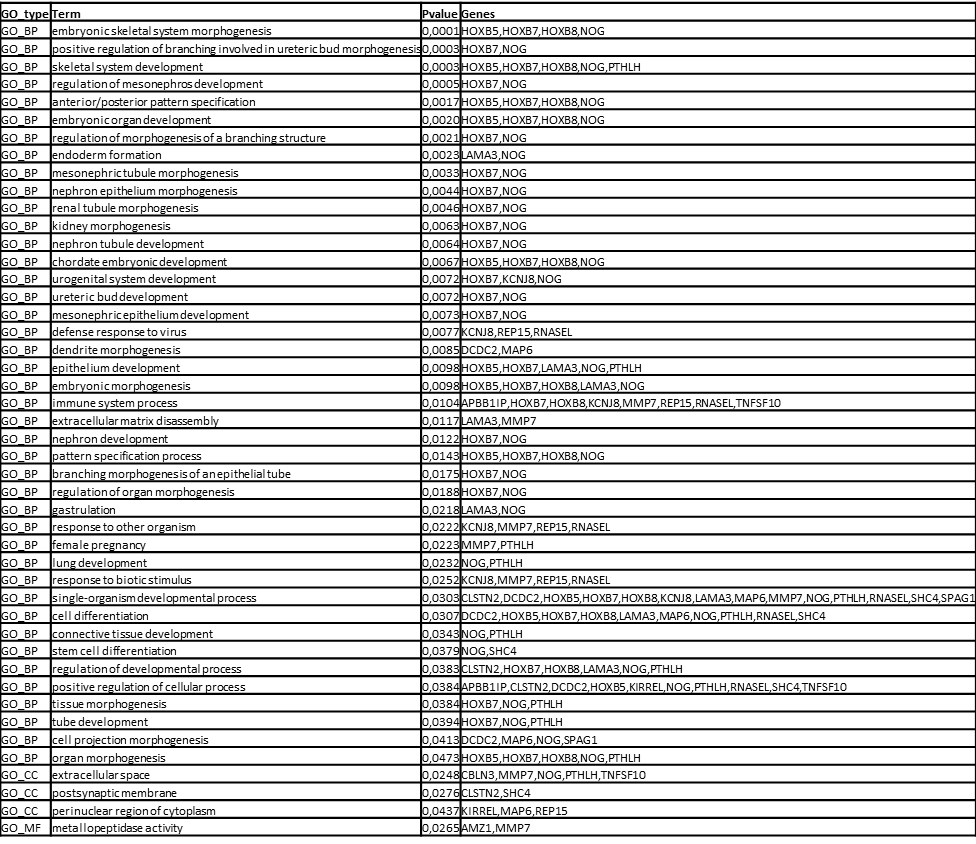

Supplement: Supplementary file 1 — The information of supplementary materials are as follows: SUPPLEMENTARY FIGURE 1: AF-MSC and pluripotent cells shared genes top 12 KEGG pathways and significant gene ontologies for biological processes and cellular components. SUPPLEMENTARY FIGURE 2: AF-MSC and fMSCs shared genes top 19 KEGG pathways and significant gene ontologies for biological processes and cellular components. SUPPLEMENTARY FIGURE 3: 181 AF-MSC-specific genes significant KEGG pathways and top gene ontologies for cellular components. SUPPLEMENTARY TABLE S1: 25 AFSC-specific genes significant KEGG pathways and top gene ontologies for cellular components specific genes from the transcriptome analysis and a public available data set from Wolfrum et al. (2010). [file 5932706.f1.docx]
