# Supplementary figures and images for: Isolation and Molecular Characterization of Amniotic Fluid-Derived Mesenchymal Stem Cells Obtained from Caesarean Sections
Source: Stem Cells Int. 2017 Oct 31;2017:5932706. doi: 10.1155/2017/5932706 (PMC5684599; doi:10.1155/2017/5932706)

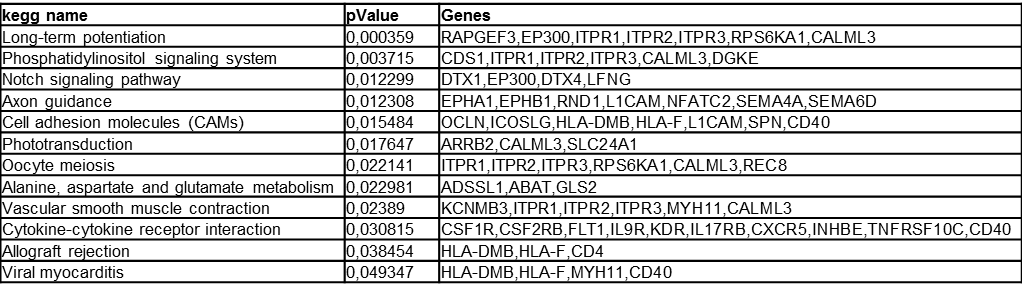


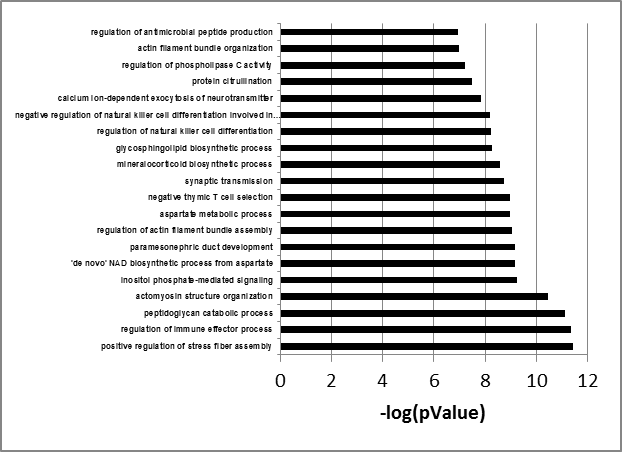

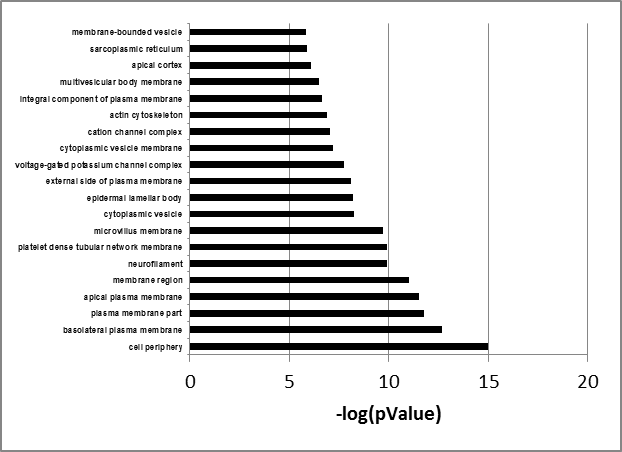


GO’s Biological Processes

GO’s Cellular Components

Supplement: Supplementary file 2 [file 5932706.f2.docx]

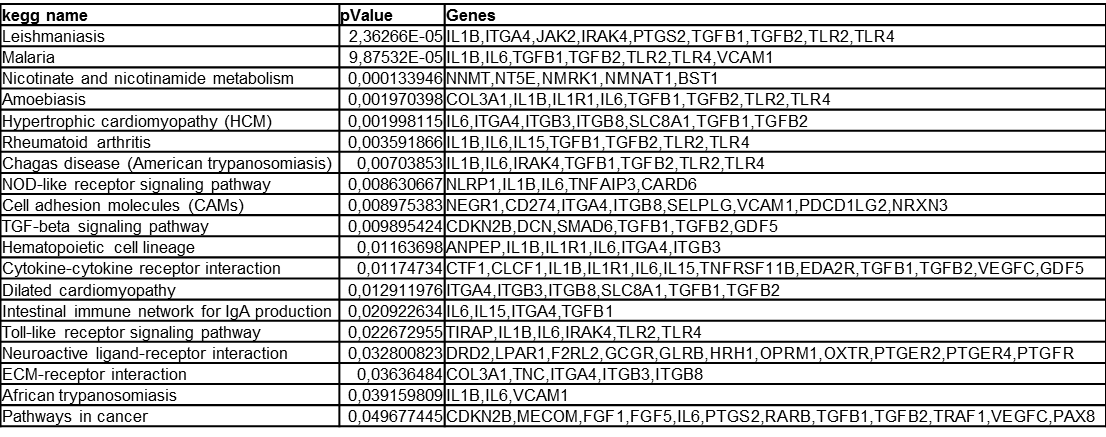


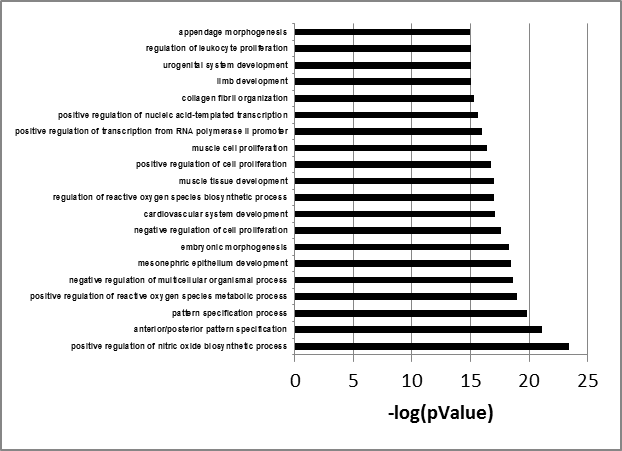


GO’s Biological Processes


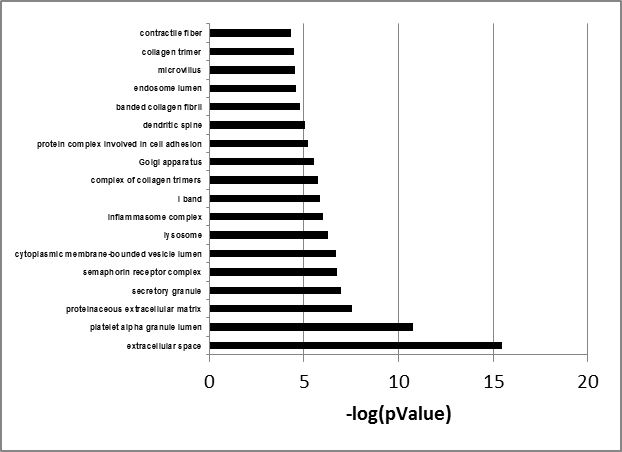


GO’s Cellular Components

Supplement: Supplementary file 3 [file 5932706.f3.docx]

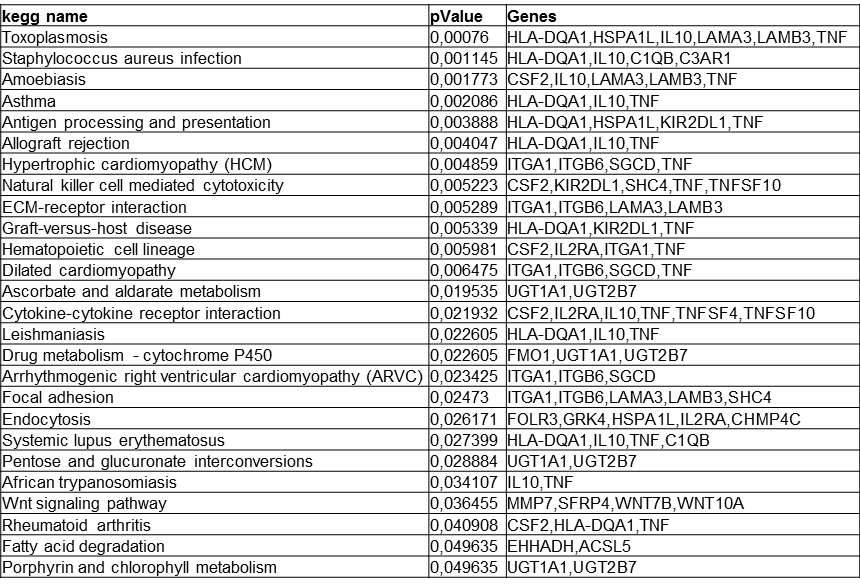


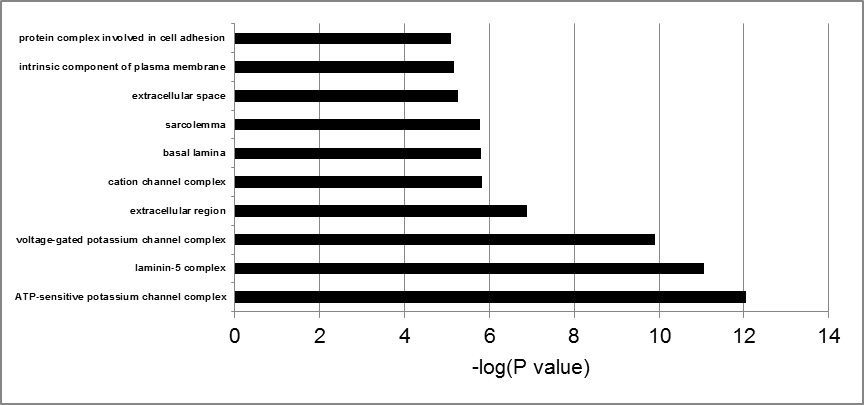


GO’s Cellular Components

Supplement: Supplementary file 4 [file 5932706.f4.docx]
