# Supplementary material for: Isolation and Molecular Characterization of Amniotic Fluid-Derived Mesenchymal Stem Cells Obtained from Caesarean Sections
Source: Stem Cells Int. 2017 Oct 31;2017:5932706. doi: 10.1155/2017/5932706 (PMC5684599; doi:10.1155/2017/5932706)
Supplement: Supplementary file 5 [file 5932706.f5.docx]

| **GO_type** | **Term** | **Pvalue** | **Genes** |
| --- | --- | --- | --- |
| GO_BP | embryonic skeletal system morphogenesis | 0,0001 | HOXB5,HOXB7,HOXB8,NOG |
| GO_BP | positive regulation of branching involved in ureteric bud morphogenesis | 0,0003 | HOXB7,NOG |
| GO_BP | skeletal system development | 0,0003 | HOXB5,HOXB7,HOXB8,NOG,PTHLH |
| GO_BP | regulation of mesonephros development | 0,0005 | HOXB7,NOG |
| GO_BP | anterior/posterior pattern specification | 0,0017 | HOXB5,HOXB7,HOXB8,NOG |
| GO_BP | embryonic organ development | 0,0020 | HOXB5,HOXB7,HOXB8,NOG |
| GO_BP | regulation of morphogenesis of a branching structure | 0,0021 | HOXB7,NOG |
| GO_BP | endoderm formation | 0,0023 | LAMA3,NOG |
| GO_BP | mesonephric tubule morphogenesis | 0,0033 | HOXB7,NOG |
| GO_BP | nephron epithelium morphogenesis | 0,0044 | HOXB7,NOG |
| GO_BP | renal tubule morphogenesis | 0,0046 | HOXB7,NOG |
| GO_BP | kidney morphogenesis | 0,0063 | HOXB7,NOG |
| GO_BP | nephron tubule development | 0,0064 | HOXB7,NOG |
| GO_BP | chordate embryonic development | 0,0067 | HOXB5,HOXB7,HOXB8,NOG |
| GO_BP | urogenital system development | 0,0072 | HOXB7,KCNJ8,NOG |
| GO_BP | ureteric bud development | 0,0072 | HOXB7,NOG |
| GO_BP | mesonephric epithelium development | 0,0073 | HOXB7,NOG |
| GO_BP | defense response to virus | 0,0077 | KCNJ8,REP15,RNASEL |
| GO_BP | dendrite morphogenesis | 0,0085 | DCDC2,MAP6 |
| GO_BP | epithelium development | 0,0098 | HOXB5,HOXB7,LAMA3,NOG,PTHLH |
| GO_BP | embryonic morphogenesis | 0,0098 | HOXB5,HOXB7,HOXB8,LAMA3,NOG |
| GO_BP | immune system process | 0,0104 | APBB1IP,HOXB7,HOXB8,KCNJ8,MMP7,REP15,RNASEL,TNFSF10 |
| GO_BP | extracellular matrix disassembly | 0,0117 | LAMA3,MMP7 |
| GO_BP | nephron development | 0,0122 | HOXB7,NOG |
| GO_BP | pattern specification process | 0,0143 | HOXB5,HOXB7,HOXB8,NOG |
| GO_BP | branching morphogenesis of an epithelial tube | 0,0175 | HOXB7,NOG |
| GO_BP | regulation of organ morphogenesis | 0,0188 | HOXB7,NOG |
| GO_BP | gastrulation | 0,0218 | LAMA3,NOG |
| GO_BP | response to other organism | 0,0222 | KCNJ8,MMP7,REP15,RNASEL |
| GO_BP | female pregnancy | 0,0223 | MMP7,PTHLH |
| GO_BP | lung development | 0,0232 | NOG,PTHLH |
| GO_BP | response to biotic stimulus | 0,0252 | KCNJ8,MMP7,REP15,RNASEL |
| GO_BP | single-organism developmental process | 0,0303 | CLSTN2,DCDC2,HOXB5,HOXB7,HOXB8,KCNJ8,LAMA3,MAP6,MMP7,NOG,PTHLH,RNASEL,SHC4,SPAG1 |
| GO_BP | cell differentiation | 0,0307 | DCDC2,HOXB5,HOXB7,HOXB8,LAMA3,MAP6,NOG,PTHLH,RNASEL,SHC4 |
| GO_BP | connective tissue development | 0,0343 | NOG,PTHLH |
| GO_BP | stem cell differentiation | 0,0379 | NOG,SHC4 |
| GO_BP | regulation of developmental process | 0,0383 | CLSTN2,HOXB7,HOXB8,LAMA3,NOG,PTHLH |
| GO_BP | positive regulation of cellular process | 0,0384 | APBB1IP,CLSTN2,DCDC2,HOXB5,KIRREL,NOG,PTHLH,RNASEL,SHC4,TNFSF10 |
| GO_BP | tissue morphogenesis | 0,0384 | HOXB7,NOG,PTHLH |
| GO_BP | tube development | 0,0394 | HOXB7,NOG,PTHLH |
| GO_BP | cell projection morphogenesis | 0,0413 | DCDC2,MAP6,NOG,SPAG1 |
| GO_BP | organ morphogenesis | 0,0473 | HOXB5,HOXB7,HOXB8,NOG,PTHLH |
| GO_CC | extracellular space | 0,0248 | CBLN3,MMP7,NOG,PTHLH,TNFSF10 |
| GO_CC | postsynaptic membrane | 0,0276 | CLSTN2,SHC4 |
| GO_CC | perinuclear region of cytoplasm | 0,0437 | KIRREL,MAP6,REP15 |
| GO_MF | metallopeptidase activity | 0,0265 | AMZ1,MMP7 |
